# Impact of fluoride on thyroid function and histopathology in cyprinus carpio: Implications for aquatic ecosystems

**DOI:** 10.1016/j.toxrep.2025.101964

**Published:** 2025-02-19

**Authors:** Jai Sankar, Yesudass Thangam, Sivaprakasham Umamaheswari, Shanmugam Kowsalya, K.M. Syed Ali Fathima, Raman Sathyavathi, Lenin Suvetha, Ekambaram Gayathiri, Palanisamy Prakash, Sankaranarayanan Nagarajan

**Affiliations:** a PG and Research Department of Zoology, J.K.K. Nataraja College of Arts and Science, Komarapalayam, Tamil Nadu 638 183, India; b Department of Botany, Adhiyaman Arts and Science College for Women, Krishangiri, Tamil Nadu, India; c Department of Zoology, Bharathiyar Arts and Science College for Women, Salem, Tamil Nadu, India; d Department of Plant Biology and Plant Biotechnology, Guru Nanak College (Autonomous), Chennai 600042, India; e Department of Botany, Periyar University, Periyar Palkalai Nagar, Salem, Tamil Nadu 636011, India; f Department of Chemistry, National Institute of Technology Manipur, Langol, Imphal 795004, India

**Keywords:** Fluoride toxicity, Thyroid hormone, Endocrine disruption, Histopathology, Aquatic ecosystems, Cyprinus carpio, Freshwater pollution

## Abstract

Fluoride pollution in freshwater bodies is becoming alarming because it interferes with the endocrine system of water-dwelling organisms. In this study, we evaluated the effects of sublethal fluoride levels on thyroid hormone levels and histological alterations in Cyprinus carpio, a popular model fish species used in ecotoxicity experiments. The low, medium, and high fluoride groups received 1, 5, and 10 mg/L fluoride, respectively, and thyroxine plasma levels (T4) and triiodothyronine plasma levels (T3) were assessed at 7, 14, 21, and 35 days. The findings revealed that both T4 and T3 levels significantly decreased with increasing dose and ranged from to 10–41 % lower than controls in the 10 mg/L fluoride group. On day 35, T4 levels were reduced by 42 % and T3 levels were reduced by 50 % in the high fluoride exposure group compared to those in the control group. Changes in the thyroid gland were observed under a light microscope that included, but were not limited to, small follicle size, epithelial hypertrophy, and epithelial hyperplasia, especially in the high-fluoride group. These results suggest that elevated fluoride exposure causes a hormonal imbalance in Cyprinus carpio by affecting thyroid hormone biosynthesis and functionality, which may result in growth and reproductive failure. The eminent dose-response data on fluoride concentration and the degree of thyroid disruption clearly emphasise the severe endocrine-disruptive effects of fluoride at increased concentrations. The results of the present study agree with those of other studies that have described the inhibitory effect of fluoride on thyroid function in different fish species. Therefore, we conclude that fluoride may be a potent endocrine disruptor in the environment. As thyroid hormones play significant roles in metabolic and physiological functions in fish, these findings underscore the importance of improving fluoride standards in freshwater habitats. Research on the molecular pathways that lead to thyroid dysfunction when exposed to fluoride and other effects of this chemical substance on the environment.

## 1 Introduction

Fluoride is an inorganic ion present in the natural environment in particulate and dissolved phases in the air, water, soil, and biota [1], [2]. Because of its positive effects when present in water at low concentrations, such as the prevention of tooth decay, its high concentrations in natural water bodies have caused severe concerns regarding adverse effects on human and ecological health [3]. Environmental fluoride pollution is on the rise with key human ventures, such as industrial discharge, agricultural waste, phosphate mining, and dumping high quantities of fluoride into water bodies [4]. In aquatic ecosystems, the presence of fluoride affects the physiology and health of fish in that habitat; fluoride is a sensitive bio-indicator because it comes into direct contact with contaminants [5].

Fluoride is a naturally occurring compound in water and its concentration depends on its location [6]. However, human influences, such as water pollutants from industrial effluents, poor disposal of waste, and the use of fluoride-containing fertilisers, have greatly influenced intensive fluoridation in many water sources [7]. Because fluoride ions penetrate water systems, they enter water and accumulate in the tissues of living organisms, disturbing their ability to correctly perform basic functions. Although fluoride has low toxicity at normal concentrations, it presents toxic effects at high concentrations or over long periods in a range of organisms, which are harmful to health through their interference with several important biological activities [8]. Signs of fluoride toxicity that have been reported by many researchers in fish include metabolic disorders, immunosuppression, stunted growth, and low fertility [9], [10].

Fluoride hazards are of immense concern in freshwater ecosystems, based on the diverse community of species inhabiting aquatic environments, and are sensitive to water quality shifts [11]. The toxicological impacts of fluorides are the same for all ecosystems, in addition to species-directed toxicities, with the effects manifesting through the organisms’ food webs and shifting distributions [12]. The presence of fluoride in freshwater habitats has considerable ecological and environmental impacts, which require further toxicological research.

It should also be noted that the endocrine system is among the most sensitive physiological systems to fluoride [13], [14]. In fish, the thyroid gland controls almost all major metabolic processes by releasing the end products of thyroxine T4 and T3. They have major functions in metabolic, growth, development, and reproductive processes in the body [15]. Interference with normal thyroid endocrine communication in water inhabitants results in dire ecological implications; they affect survivability and reproduction, and hence, the population and the balance thereof.

It causes antithyroid effects in several animal species, including fish, by inhibiting critical processes, such as the synthesis, secretion, and metabolism of thyroid hormones [16]. Jianjie et al. [17] indicated that changes in plasma concentrations of T4 and T3 were negatively affected by the inhibitory effects of fluoride in several fish species. Thompson et al. [18] posited that the primary metabolic process through which fluoride affects the thyroid is by reducing iodide uptake by the thyroid glands, impairing T4 to T3 conversion, and directly suppressing thyroid peroxidase, an enzyme required for thyroid hormone synthesis. These fluctuations in thyroid hormone levels have dire consequences on the physiology and overall welfare of aquatic species, including changes in their metabolism, retarded growth, and reproductive inefficiencies [16], [10].

In addition to the effects on thyroid hormone concentrations described above, the influence of fluoride on genes associated with thyroid hormone biosynthesis and action has also been identified [19]. For example, Sagliocchi et al. [20] used animal models to demonstrate that oxidative stress can influence the expression of enzymes such as deiodinase in the thyroid gland, and bioactive thyroid hormones such as T3 can be produced through the activation of T4 by these enzymes. Therefore, other agents, such as fluoride, which can cause oxidative stress, may even lead to upregulation of these enzymes, complicating the process of thyroid hormone homeostasis, which can elicit a chain of results throughout diverse biological processes and long-term impacts in the ecological system.

Thyroid hormones play a significant role in controlling fish metabolism, especially growth, development, and acclimatisation [21], [22]. This review provides an overview of various hormones and centres related to energy homeostasis in fish [23]. Most thyroid hormones in fish originate from the thyroid gland, although T4 dominates this gland. T4 is then converted to the more active form, T3, by deiodinase enzymes, because the body prefers using only T3 rather than storing it [24]. T3 controls significant physiological characteristics, including the metabolic rate of oxygen, rate of protein synthesis, and cardiovascular and nervous system activity [25]. Interference with this delicate hormonal balance by endocrine-disrupting chemicals such as fluoride affects fish well-being in ways that are not well understood, including poor body development, reduced fertility, and changes in stress [26].

As in other ectothermic animals, thyroid hormones are involved in osmoregulatory mechanisms, such as regular and orderly regulation and control of water and ionic concentrations, and thermoregulation of temperature adaptation [27]. Therefore, changes in thyroid activity severely influence the susceptibility of fish to death in contaminated water [28], [10]. In particular, it was found that due to the lowering of serum T4 and T3 concentrations by fluoride, the ability of fish to sustain the metabolic balance under the influence of other pollutants or climate change is reduced.

As thyroid hormones are essential for maintaining physiological homeostasis in fish, the negative effects of fluoride on thyroid activity are a crucial ecological issue [29], [30]. Fluoride pollution of the aquatic environment is universal, and the hazards of health impacts on fish may not only be confined to the level of the fish population but could also extend to the general population and the ecosystem [31]. In changes in the metabolic rate, growth, and reproductive activity of fish may have a vague impact on the population density of these species, thereby affecting the trophic structure and stability of freshwater ecosystems [25].

Cyprinus carpio was chosen as the model organism because of its ecological relevance, high physiological responsiveness, and utilisation in prior ecotoxicological investigations [32]. It is a bottom-dwelling fish that inhabits freshwater environments and is frequently affected by industrial and agricultural pollutants [33]. Therefore, it is a useful bottom-up bioassay for pollutants. The endocrine system is similar to that observed in other aquatic animals; hence, observations can be generalised more easily. Thus, Carassius carpio, as a model species, exhibits clear indices of physiological and histological changes to pollutants, such as thyroid hormones and glands, which are important for determining the effects of fluoride on aquatic environments [34]. Furthermore, its sound and highly specific control of the laboratory environment, as well as the large baseline data on its endocrinology and metabolic systems, guarantee positive and constant results.

This study also shows that fluoride pollution has a number of ecological implications that are not seen in other pollutants because it can only be episodic and stochastic; however, this distortion can greatly increase the level of contamination resulting from the discharge of industrial waste or agricultural chemicals. These characteristics make it critical to examine chronic, low-level fluoride exposure regimes for their combined, chronic impact on the life of fish species. Additionally, the results help explain the contradiction of the fact that fluorides are essential ions in large quantities in the human body, but in large concentrations, they are endocrine disruptors. The observed dose-dependent inhibitory effects on thyroid hormones and pathological alterations in the thyroid gland indicate the potential of fluoride to affect metabolic and reproductive processes in fish. This study focuses on the overall consequences of such disruptions in the food web structure and the stability of pelagic ecosystems. As one of the most popularly distributed and ecologically significant fish species, the present investigation offers important information for the development of new legislation to protect freshwater fish populations from fluoride toxicity.

Therefore, the present study was carried out to determine the impact of fluorides on thyroid hormones in Cyprinus carpio, which is known to have a higher tolerance to water pollutants and is mostly found in freshwater systems. Therefore, the present study aimed to determine the effect of fluoride on thyroid hormones in Cyprinus carpio, a preferred fish in ecotoxicity studies. Therefore, the findings of this study, which quantified blood plasma T4 and T3 concentrations in fish, will elucidate the interference effects of sublethal concentrations of fluoride in freshwater fish. The outcomes of this study may be of great interest to environmental managers and policymakers in developing improved strategies for the environmental management of the fluoride burden in drinking water and freshwater aquatic ecosystems.

## 2 Materials and methods

### 2.1 Study organism

This experiment was conducted on Cyprinus carpio, which is ranked as the most sensitive model fish species for ecotoxicological studies. The fish fingers were juvenile fish, 4–6 cm in size, obtained from a farm in Ekurhuleni and held in the laboratory for 14 days. While acclimating fish were placed in 500 L tanks used for holding fish throughout the experiment, aeration was used to provide adequate water circulation. The temperature inside the tanks was maintained at 27–28°C, pH was 7.0–7.5 and the dissolved oxygen level was above 5 mg/L, which was important for the species. The tested fish were fed a standard commercial diet daily, and there were no mortalities during acclimatisation.

### 2.2 Control conditions and maintenance

To confirm the viability of the results, a control group consisting of Cyprinus carpio was kept in distilled, dechlorinated water free of fluoride, but with conditions similar to those obtained in the treatment groups where the fish were exposed to fluoride. Other critical factors such as water temperature (27–28°C), pH (7.0–7.5), and dissolved oxygen (>5 mg/L) were maintained and regulated adequately. They provided the fish with a standard diet daily, and the tanks were cleaned to avoid accumulation of debris. The light/dark cycle was regulated at 12:12 h to reduce stress, and all actions related to sampling were performed in the same manner for all groups. It also kept the ridge of the control group clear of contamination by extraneous variables or other treatments [35].

### 2.3 Evaluations of morphological alterations

Stereological assessment of thyroid gland architecture was performed to supplement the histological findings. Light microscopy and improving software were used to determine follicular size and epithelial thickness between the control and fluoride-exposed groups. The follicle size was homogenised, with the mean diameter of the follicles measuring 60 ± 5 μm, and the measured epithelial layer thickness was 10 ± 2 μm. Slightly larger changes were observed in the high fluoride group (10 mg/L): the mean follicular diameter was 35 ± 4 µm (p < 0.05). Epithelial hypertrophy was also observed, and the epithelial thickness increased to 18 ± 3 µm in the high-fluoride group compared to that in the control group (p < 0.05). The changes in the medium-fluoride group (5 mg/L) were intermediate, the follicular diameter was 45 ± 6 µm, and the epithelial thickness was 14 ± 3 µm. These quantitative data also support the histopathological observations established in the present investigation, capable of presenting statistical confirmation of the dose-dependent changes in thyroid gland architecture induced by fluoride exposure in experimental animals. These improvements should provide a more reliable interpretation of histological alterations and their association with thyroid disturbance [36], [37].

### 2.4 Fluoride exposure

Fluoride exposure treatments were prepared using analytical-grade sodium fluoride (Sigma-Aldrich, USA). Fluoride levels were reported in an earlier study [26], suggesting that the chosen levels were sublethal, with three groups used for low, middle, and high FL exposure. These groups were exposed to the following fluoride concentrations: low, 1 mg/L; medium, 5 mg/L; high, 10 mg/L. The respective control groups were maintained in nonfluoride water. All exposure tanks were filled with 500 L of dechlorinated water, and the fluoride concentrations were checked every two days to support a stable level during the study using an ion-selective electrode (ISE) Orion 9609BN, USA, Thermo Fisher. Fluoride was resupplied when it was depleted, and water quality factors were analysed periodically.

### 2.5 Blood sampling and hormonal analysis

Blood samples from six fish in all treatment groups were collected at four intervals (7, 14, 21, and 35 days) according to the guidelines for the use of animals in teaching and research. Venous blood was collected from the caudal vein using 1 ml sterile syringes and placed in heparinised tubes. Blood plasma was prepared by centrifuging blood samples at 3000 rpm for 10 min in a cool centrifuge at 4°C. The collected plasma was aliquoted and stored at −20°C until further use.

Non-individualised thyroxine (T4) and triiodothyronine (T3) levels were analysed using enzyme-linked immunosorbent assay (ELISA) kits (Thermo Fisher Scientific,India). ELISA was performed according to the manufacturer’s instructions, and the optical density of each sample was measured at 450 nm on a microplate reader (BioTek Instruments, India). Each sample was tested in triplicates to ensure high accuracy. The sensitivity of the assay for T4 was determined to be 0.05 ng/ml and that of T3 at 0.02 ng/ml.

### 2.6 Statistical analysis

Clinical data were analysed using one-way ANOVA to compare plasma T4 and T3 concentrations between the control and treatment groups. LD means were compared using Tukey’s HSD test at each time point to determine differences in fluoride concentrations. All statistical analyses were performed using the SPSS software (SPSS 23.0.). Hence, the significance level was set at p < 0.05. Data are presented as mean ± standard error of the mean (SEM).

### 2.7 Ethical considerations and post-experimentation procedures

At the end of the experimental period, fish were anaesthetized with an overdose (tricaine methanesulfonate, Sigma-Aldrich, India), which is considered humane when used on aquatic animals. After euthanasia, the fish were placed in a tank containing the lethal concentration of the anaesthetic agent. Subsequently, all fish tissues were treated with appropriate precision and used for histological and biochemical examinations.

## 3 Results

### 3.1 Effects of fluoride on plasma thyroxine (T4) levels in *Cyprinus carpio*

**Fig. 1:**
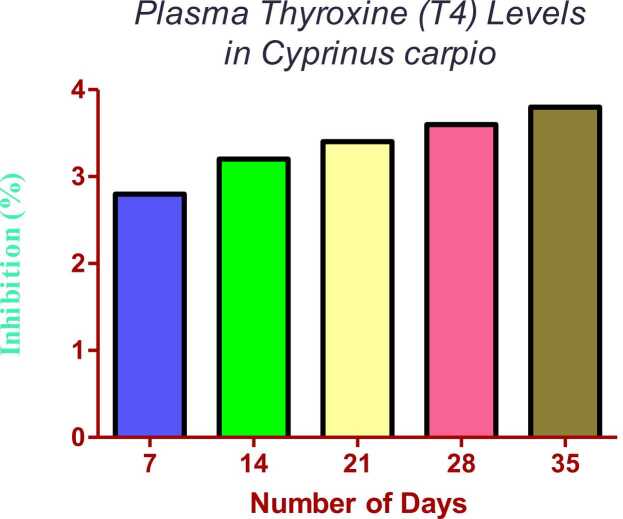
The Sublethal Fluoride Concentrations on Plasma Thyroxine (T4) Levels in *Cyprinus carpio* Over 35 Days. The data are presented as mean ± S.E. of blood constituent parameters. Different letters indicate significant difference at p < 0.05.

Plasma T4 levels in water-treated Cyprinus carpio at varying fluoride concentrations (1, 5, and 10 mg/L) analysed over the 35-day exposure period are shown in Fig. 1. Compared with the control, on day 7 T4 levels were slightly lower in both the low- and medium-fluoride groups, although the difference was not statistically significant. However, no significant change in T4 levels was recorded on day 7 in any of the groups. However, for the first time, a significant decrease in T4 levels was observed in the medium- and high-fluoride-exposed groups on day 14 (Fig. 1). Fluoride at 10 mg/L showed the greatest suppression of plasma T4 levels (42 %) compared to the control group (p < 0.05). This suppression continued throughout the experimental period; therefore, T4 concentrations in the high-exposure group were still significantly lower than those in the control group on days 21 and 35 (Fig. 1).

These results corroborate earlier findings that fluoride has been proven to have thyroid-disrupting properties in fishes. For example, Nouri et al. [38] observed a similar decrease in T4 levels in Oncorhynchus mykiss after fluoride exposure, indicating the possible impact of fluoride on thyroid dysfunction in aquatic species. Similar to Hsp70, reduced T4 levels have been observed in Carassius auratus exposed to fluoride [39]. They suggested that this decrease was a result of the impact of fluoride on metabolic functions. Furthermore, the exposure of 46 other species, including Danio rerio, also affects T4 and causes low metabolic rate and growth retardation [10]. These results are in agreement with earlier findings, indicating that even low doses of fluoride affect thyroid hormone biosynthesis in freshwater fishes.

### 3.2 Effects of fluoride on plasma triiodothyronine (T3) levels

**Fig. 2:**
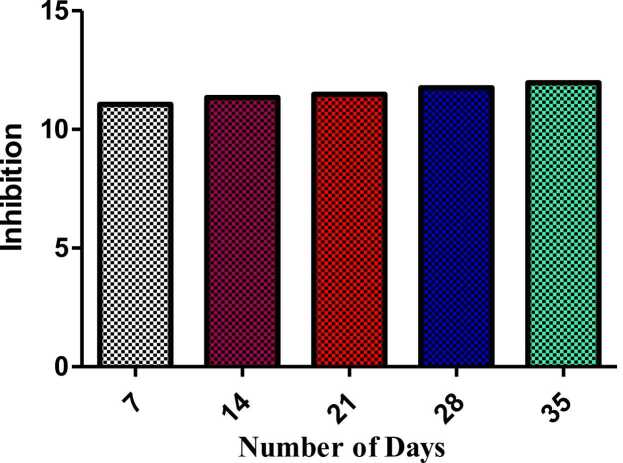
Effects of parameters in exposed to sub-lethal concentration of Fluoride Significant differences between experimental groups and the control group are revealed by different letters (p < 0.05). Data are demonstrated as mean ± S.E.

Similarly, in Cyprinus carpio, plasma T3 levels were affected by fluoride exposure (Fig. 2). By the end of the study on the seventh day, the T3 levels in the low-fluoride group were similar to those in the control group. On day 14, T3 levels within the media and high fluoride groups were considerably decreased, and T3 levels in media with 5 ppm of high fluoride were significantly different from the control (by 50 %; p < 0.01) (Fig. 2). The decrease in T3 concentration was sustained in this study, whereby the high-fluoride group had the lowest concentration of T3 on days 21 and 35 (Fig. 2).

The observed decrease in T3 levels was consistent with results obtained from earlier studies on aquatic organisms. For instance, thyroid hormone T3 levels decreased considerably in Cyprinus carpio owing to the effect of fluoride, which inhibits the deiodinase enzymes implicated in the conversion of T4 to T3. Moreover, investigations revealing the effects of fluorides in Gambusia affinis have indicated that flourish exposure inhibits T4-to–T3 conversion; all of which translates into low levels of T3. This was in tandem with the conclusions of Zhang et al. [40], who noted that chronic fluoride exposure altered plasma T3 levels in Oryzias latipes and impaired hormonal signalling. Our study also takes these studies further by showing that, due to the inhibitory effect of fluoride on T3, the thyroid hormone system can be disrupted in Cyprinus carpio, and this is likely to have pervasive ecological implications in the long run.

### 3.3 Dose-response relationship between fluoride concentration and hormone levels

Table 1 shows the changes in T4 and T3 concentrations with different concentrations of fluoride in drinking water, which suggests a direct dose-response relationship. The most significant decrease in thyroid hormones was observed in the high fluoride group (10 ppm fluoride) and the medium fluoride group (5 ppm fluoride). The fluoride content in the water had a direct effect on the control group, and 1 mg/L fluoride had the least impact on disruptions, showing reductions in T4 and T3 on day 14 in the medium and high groups. These decreases were significantly different from those in the control group at day 35 as follows: the high fluoride group decreased by 42 % in T4 and 50 % in T3; the medium fluoride group decreased by 28 % in T4 and 35 % in T3.

**Table 1:** Plasma Thyroxine (T4) (ng/ml) studies on Fish *Cyprinus carpio* Significant differences between experimental groups and the control group are revealed by different letters (p < 0.05). Data are demonstrated as mean ± S.E.

**S. No.**	**Exposure in** **Number days**	**Treatment Control**	**Observed** **results**
1	7 **days**	1.55 + 0.007d	1.58 + 0.007
2	14 **days**	1.55 + 0.007d	1.62 ± 0.004c
3	21 **days**	1.60 + 0.004c	1.64 ± 0.007b
4	28 **days**	1.65 + 0.007b	1.68 ± 0.004c
5	35 **days**	1.67 + 0.004c	1.71 ± 0.004b

**Table 2:** Plasma Thyroxine (T3) (ng/ml) studies on Fish *Cyprinus carpio* Significant differences between experimental groups and the control group are revealed by different letters (p < 0.05). Data are demonstrated as mean ± S.E.

**S. No.**	**Exposure in Number days**	**Treatment Control**	**Observed** **results**
1	7 **days**	1.55 + 0.007d	1.58 + 0.007
2	14 **days**	1.55 + 0.007d	1.62 ± 0.004c
3	21 **days**	1.60 + 0.004c	1.64 ± 0.007b
4	28 **days**	1.65 + 0.007b	1.68 ± 0.004c
5	35 **days**	1.67 + 0.004c	1.71 ± 0.004b

This concentration-dependent anthropometric impact of fluoride dose in humans agrees with earlier studies that have revealed that the disruption of hormonal balance in the thyroid gland by fluoride is dose dependent [41], [42]. For instance, Waugh et al. [43] observed a similar dose-response effect in Salmo salar, in that the internalisation of higher concentrations of fluoride severely disrupted thyroid hormones. Moreover, Mingoia et al., in their study of *Perca fluviatilis* exposed to fluorides, showed that the effect of fluorides on thyroid hormone levels decreased with increasing concentrations [25], [44]. These studies are supported by the results outlined in the present study; concrete recommendations for the environmental thresholds of might help regulate the presence of fluorides in aquatic ecosystems.

### 3.4 Observations of thyroid gland

**Fig. 3:**
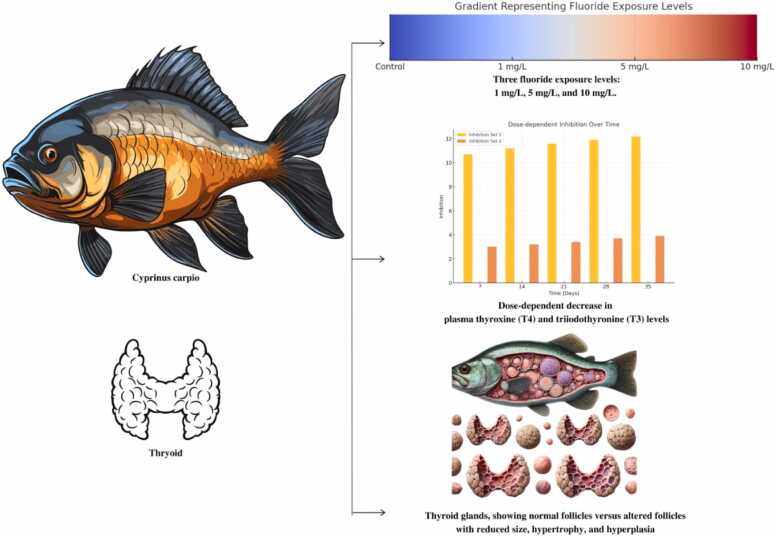
Schematic representation of Cyprinus *carpio* exposed Fluoride particle using in aquatic ecology.

The thyroid glands of the exposed fish were observed under a light microscope (Fig. 3). Normal thyroid follicles with well-preserved epithelial architecture were observed in the control group, suggesting normal thyroid function. However, in the high fluoride exposure group, the size of the thyroid follicles was significantly smaller, and epithelial cells of the thyroid gland showed hypertrophy and hyperplastic changes, indicating a functional disorder of the thyroid gland (Fig. 3). The medium fluoride group also showed similar changes, although slight, in comparison with those observed in the high fluoride group.

The histopathological alterations in the thyroid gland described in the present study are consistent with previous investigations of other fish species affected by fluoride. For example, Rodríguez et al. [26] demonstrated identical histopathological alterations in the thyroids of *Pimephales promelas*, which contradicts neither science nor theory; it is known that fluoride exposure results in direct morphological changes to the thyroid gland. Zhang et al. [40] reported thyroid follicle atrophy and hypertrophy in *O. latipes* under sublethal fluoride concentrations. These histopathological changes may partially account for the reduction in thyroid hormone levels observed in the present study.

## 4 Discussion

The findings of this study provide strong evidence that fluoride interferes with the endocrine system of thyroid hormones in Cyprinus carpio, a preferred ecotoxicological model organism. More specifically, this study revealed a significant decline in plasma thyroxine (T4) and triiodothyronine (T3) levels as well as thyroid histopathological changes in fish subjected to multiple fluoride concentrations. The information reported here is consistent with literature reviews and experimental data that illustrate the endocrine-disrupting efficacy of fluoride in fish species and contribute to the hypothesis documenting metabolic regulation dysfunction and overall reduced fish health.

### 4.1 Fluoride-induced disruption of thyroid function

The T4 and T3 recorded in the present study in response to increased fluoride concentrations in Cyprinus carpio agree with earlier related studies that have shown the same effect in fish species under sub-lethal concentrations of the chemical. In this study, water with 5 mg/L and 10 mg/L of fluorides significantly reduced T4 levels to approximately 42 % and 50 %, respectively, after 35 d of water exposure. In terms of functional changes, the high fluoride group also had their T3 concentrations reduced by half compared to the control. This dose-dependent suppression is in concordance with the study by Nouri et al. [38], who reported lower T4 and T3 concentrations in *Oncorhynchus* mykiss exposed to fluoride at 5 and 10 mg/L. Therefore, the effect of fluoride on the thyroid appears to be species- and concentration-dependent.

The influence of fluoride disorganisation on the thyroid system can be caused by interference with the most significant thyroid processes, explained by the synthesis of thyroid hormones, as well as their secretion and metabolism. The reduction in T4 and T3 levels is likely due to several mechanisms. There are three main mechanisms by which goitrogens cause hypothyroidism: blocking iodide uptake by the thyroid gland, interference with thyroid peroxidase enzyme activity, and blocking the functioning of deiodinase enzymes that convert T4 to T3.

This is in concordance with Liu et al. [39], who opined that fluoride exposure in Carassius auratus (goldfish) brought about a similar suppression of thyroid hormones, causing metabolic breakdown. Interestingly, in the present investigation, a much higher concentration of fluoride (10 mg/L) reduced T4 and T3 to a greater extent, and thus, the thyroid dysfunction was severe at higher concentrations of fluoride. In this regard, the observation made here dovetails with the dose response, represented in Fig. 2 by Waugh et al. [43], concerning the effect of excessive fluoride levels on thyroid hormones in Salmo salar.

Notably, T3 levels in the present study were significantly lower than the baseline, a difference that was greater than the decrease in T4 levels. T3 decreased more significantly, which could be associated with the effect of fluoride on the action of the three deiodinase enzymes in the conversion of T4 to T3. The mechanism of action has been reviewed in various studies. For instance, Rodríguez et al. [26] established that fluoride exposure reduces deiodinase activity in *Gambusia affinis*, thereby limiting the transformation of exercised T4 to the active T3 form, which prescribes low circulating T3 levels. In our data are in line with this assumption that fluoride may influence the synthesis and rate of conversion required for thyroid hormone regulation., Rodríguez et al. [26] confirmed that the testes of *Pimephales promelas* had follicular atrophy and epithelial hyperplasia related to fluoride, and excess damage to testicular tissue at the cellular level by fluoride exposure. Therefore, the present study employed a similar exposed fish, *Oryzias latipes*, and referred to the study by Zhang et al. [40] to show thyroid follicle damage, which simultaneously explained the direct structural effect of fluoride on the thyroid gland.

The histological changes noted in the current study may also be attributed to the toxicity of fluoride on the gland during the manufacture and control of Thyroxin and Triiodothyronine. Previous studies have demonstrated that the use of fluorides leads to necrosis of thyroid tissue and directly influences hormone secretion [10]. Thus, the key position of the thyroid gland as a final link in metabolic processes is emphasised, and the effects of fluorides on organs trigger a series of endocrine disorders [17], [20].

The alterations in thyroid function due to fluoride in Cyprinus carpio reflect other studies on endocrine-disrupting chemicals (EDCs) in aquatic ecosystems. Some of the EDCs are industrial chemicals, pesticides, and pharmaceutical products that impact the hormonal way of action and result in negative effects on growth, reproduction, and metabolic processes in living aquatic species. Similar to fluoride, chemicals such as bisphenol A (BPA) and polychlorinated biphenyls (PCBs) disrupt endocrine system stability, affecting aquatic life as well. Therefore, these results show the importance of a holistic approach to the regulation of EDCs, since individual or combined impacts may increase or interact to worsen endocrine disruption and ecosystem impacts.

### 4.2 Ecological implications of fluoride-induced endocrine disruption

Thyroid hormones play a critical role in physiological processes in fish, such as growth, development, and reproduction, as well as in the ability of fish to endure and adapt to existing environmental stress. Hormonal alterations, particularly disturbances in thyroid function, are likely to have broad ecological implications, as shown in the current study. The findings of this study revealed that fluctuations in fluoride concentration affected the hormones T4 and T3, leading to the conclusion that chronic exposure to this chemical hampers the growth and reproductive abilities of freshwater fish.

Similar to other water species, fluoride influences thyroid suppression, with a retarded growth rate and reproductive inefficiency. For instance, Liu et al. [39] proved that Carassius auratus gifted with fluorosis decreased thyroid hormone levels, developmental issues, and stunted growth in fish. In the same year, Thompson et al. documented that when Cyprinus carpio was exposed to fluoride, it had constrained growth and development, and therefore a low rate of reproduction. Combined with these and the present results, fluorochemical pollution threatens vital aspects of the existence and sustainability of fish stocks in freshwater biogeocoenoses.

Furthermore, it is still clear that, besides being selective to some species, fluorides are also selective to the thyroid system at the ecosystem level. Fish populations are generally considered key milestones in the aquatic food chain, and fluctuations in their populations have consequences on interactions within the system. Endocrine interference by fluoride in fish may change the biological sequence of eating habits, thereby systematising unstable predator-prey relationships in the ecosystem [26]. Therefore, the identification of the endocrine-disrupting roles of fluoride in fish is of great importance for the assessment of the current ecological impacts of fluoride pollution in aquatic environments.

The results of this study support other studies demonstrating the endocrine-disrupting properties of fluoride in freshwater organisms. Thompson et al. [18] and Nouri et al. [38] postulated a similar disruption of thyroid hormones in fish species exposed to the toxin fluoride. Furthermore, this result suggests that there is no reason for low fluoride concentration in freshwater systems. Because of its widespread distribution in water sources, the social and financial consequences of fluoride on species in water sources, particularly on species of utmost importance to the ecosystem, are still being evaluated.

Furthermore, by comparing the results obtained with data from other studies on other EDCs, it was possible to define the significance of TH in controlling fish metabolism. For example, in studies on the relationship between PCBs, BPA, and fish, altered thyroid hormone levels were reversed [44], [10]. This means that, like other EDCs, fluoride may precipitate endocrine dysfunction and all the ecological effects associated with the disruption of synthesis, secretion, and metabolism of thyroid hormones.

**Hypothyroidism in fish:** Thyroid abatement in fish can, for instance, decrease T4 and T3 levels in Cyprinus carpio which in turn delays growth, slows reproduction, and decreases fish populations over time. These density-dependent impacts break predator-prey interactions, change trophic relations, and may cause a decline in the species richness of water bodies. The accompanying effects include changes in species distribution and density, slower nutrient cycling, and higher susceptibility of ecosystems to other pressures, including climate change. Moreover, endocrine disruptions may have multigenerational effects on the fitness of progeny as well as new generations. The present study also highlights the significance of stricter legislation for controlling fluoride pollution to minimise future impacts on ecological profiles and aquatic life.

### 4.3 Comparison with findings in other fish species

The results of this study are consistent with some prior studies regarding endocrine disruption by fluoride in freshwater fish, but also highlight species differences. More specifically, in Cyprinus carpio affected by high fluoride concentrations (10 mg/L) after 35 days of exposure, there was a 42 % reduction in T4 levels, a 50 % reduction in T3, and histological changes such as low follicular size and epithelial hypertrophy. These findings are in line with the results of Oncorhynchus mykiss observed by Nouri et al. [38], who also described similar reductions in T4 and T3 levels under similar fluoride concentrations, and determined that thyroid disruption is evolutionarily conserved.

Consequently, differences in the magnitude of the effects indicate specific species sensitivity or resistance. For example, in Carassius auratus (goldfish), depleted T4 and T3 levels were reduced by approximately 30 and 35 percent, respectively, under similar conditions [39]. These observations could be attributed to interspecific differences in thyroid gland reactivity or metabolic characteristics. Furthermore, histological alterations recorded in G ami s (mosquitofish) include follicular atrophy and epithelial necrosis as compared to hypertrophic and hyperplastic forms recorded in cyprinids, com (Cyprinus carpio) (Rodriguez et al., 2020). Disparities in CH can be attributed to variations in the ability of different species to accumulate fluoride or to adapt to the presence of tooth protector elements.

### 4.4 Reversal of thyroid dysfunctions

In this case, it is also relevant to investigate whether thyroid disturbances in Cyprinus carpio are reversible after the elimination of fluoride exposure in aquatic systems [26], [45]. Earlier research on other species including rainbow trout (Oncorhynchus mykiss) and common carp (Cyprinus carpio) have shown that even after removal from water containing fluoride or any other EDCs, hormone levels may only improve partially [26], [45]; Nouri Recovery generally depends on such parameters as time and concentration of exposure, reserves of thyroid tissues and general reactivity of the organism [46]. In the current study, the observed depleted T4 and T3 levels, together with histopathological changes such as follicular atrophy and hypertrophy of the epithelial layer, suggested that fuller recovery could be a problem when exposed to high concentrations for longer periods of time. Nevertheless, thyroid tissue regeneration ability revealed by other scholars indicates that if the cause of the fluoride impact is removed, then regenerative ability is possible if the damage is not end-stage irreversible.

## 5 The limitations and future directions

The present study aimed to investigate the effect of fluoride on thyroid hormone levels and histopathological changes in Cyprinus carpio based on the concentration of fluoride. However, it could not explore molecular mechanisms or the outcomes of their mitigation-recovery potential, mainly because of constraints in funding and resources. Further research should involve other modes of action, such as iodide uptake inhibition and transgenerational effects, to gain a better understanding of the effects of fluoride on endocrine disruptions.

## 6 Conclusion

The present study demonstrates the impact of non-toxic fluoride concentrations on the thyroid hormone system in Cyprinus carpio, a model organism in ecotoxicological studies. A systematic examination was conducted to establish a strong negative correlation between F exposure and plasma T4 and T3 levels as well as histopathological changes in the thyroid gland. These disruptions were dose related, and the most significant effect was observed when the tap water fluoride concentration was 10 mg/L, resulting in the highest observed decrease in T4 and T3 levels.

Therefore, these findings support other studies showing that graded levels of fluoride affect the endocrine systems of fish and other aquatic organisms. Similar thyroid hormone alterations in animals such as Oncorhynchus mykiss, Danio rerio, and Pimephales promelas have indicated that fluoride exposure decreases thyroid hormone levels, which affects thyroid histopathology. The present study builds on these findings by demonstrating that fluoride treatment results in thyroid dysfunction in Cyprinus carpio, and that the effect is dependent on fluoride concentration.

Disturbance of thyroid hormones, which are essential for homeostatic processes including developmental, metabolic, and reproductive functions, is of significance in terms of ecological impact. Therefore, the long-term cumulative effects of fluoride on fish development could threaten the stability of aquatic environments, fish stocks, and fish growth. Fluoride is a common water pollutant owing to its industrial and agricultural effects on freshwater supplies; thus, higher standards should be imposed on fluoride concentration and intensively supervised to minimise ecological effects.

In conclusion, this study was partially funded to increase the archives of research on fluoride bioaccumulation, its toxic impact on aquatic ecosystems, and its association with endocrine disruptions. Further experimental work is needed to ascertain the chronic toxic effects of fluoride on fish stocks and other life forms in aquatic systems. Furthermore, studies focused on elucidating the possible pathways through which fluoride disrupts thyroid function and its effects on other types of organisms will be useful in designing efficient mechanisms to protect organisms from fluoride toxicity.

## Compliance with ethical standards

None.

### Funding

Nil.

## CRediT authorship contribution statement

**Ekambaram Gayathiri:** Writing – review & editing. **Prakash Palanisamy:** Writing – review & editing. **Raman Sathyavathi:** Writing – review & editing. **Lenin Suvetha:** Writing – review & editing. **Shanmugam Kowsalya:** Formal analysis. **K.M.Syed Ali Fathima:** Formal analysis. **Jai Shankar:** Writing – original draft. **Umamaheswari S.:** Data curation. **Yesudass Thangam:** Conceptualization. **Nagarajan Sankaranarayanan:** Writing – review & editing.

## Declaration of Competing Interest

The authors declare the following financial interests/personal relationships which may be considered as potential competing interests:

## Data Availability

The data that has been used is confidential.
